# The Influence of Solid Content Distribution on the Low-Field Nuclear Magnetic Resonance Characterization of Ferric-Containing Alkali-Activated Materials

**DOI:** 10.3390/ma19020272

**Published:** 2026-01-09

**Authors:** Zian Tang, Yuanrui Song, Wenyu Li, Lingling Zhang

**Affiliations:** 1 School of Energy and Environmental Engineering, University of Science and Technology Beijing, Beijing 100083, China; m202410188@xs.ustb.edu.cn (Y.S.); zhanglingling@ustb.edu.cn (L.Z.); 2 State Key Laboratory of Iron and Steel Industry Environmental Protection, University of Science and Technology Beijing, Beijing 100083, China; 3 Department of Chemistry, Tsinghua University, Beijing 100084, China

**Keywords:** alkali-activated material, nuclear magnetic resonance, magnetic resonance imaging, bleeding water, slurry stratification

## Abstract

**Highlights:**

**What are the main findings?**

- Paramagnetic or ferrimagnetic particles and excess water distort LF-NMR analysis.
- Layered analysis reveals settling creates vertical signal gradients.
- A screening method is proposed to ensure sample homogeneity.

**What are the implications of the main findings?**

- Settled slurry top layer has higher free water and fewer paramagnetic substances.
- Preventing slurry settling ensures reliable LF-NMR interpretation; BFS stable, FA stratified.
- Slurry must be vertically homogeneous in water for reliable LF-NMR testing.

**Abstract:**

Recent applications of low-field NMR in alkali-activated materials (AAMs) often adopt interpretation models developed for Portland cement systems, overlooking the distinct influences of paramagnetic/ferrimagnetic components and free-water redistribution. This study investigates how paramagnetic or ferrimagnetic component and free water distribution influence low-field nuclear magnetic resonance (LF-NMR) and proton density magnetic resonance imaging (PD-MRI) characterization of alkali-activated materials (AAMs). Blast furnace slag, fly ash, and steel slag were activated with NaOH solution at liquid-to-solid ratios of 0.45 and 0.5, and analyzed across top, middle, and bottom layers. Slurries prepared with less mixing water and CaO-rich raw materials exhibited negligible settling and uniform relaxation behavior, whereas those with higher water content and CaO-deficient raw materials showed pronounced stratification, resulting in distinct gradients in signal intensity. The results indicate that the LF-NMR data interpretation of relatively dilute system may be unreliable as the relaxation time of protons will be extended after they transfer from bottom to the top of the slurry. A preliminary method for assessing slurry suitability for LF-NMR characterization is proposed for future validation.

## 1. Introduction

Recent advancements in understanding the relationship between proton relaxation and water mobility have established low-field nuclear magnetic resonance (LF-NMR) relaxometry as a powerful tool for characterizing water states in cementitious systems. LF-NMR relaxometry offers a reliable, user-friendly, and non-destructive method capable of real-time and continuous monitoring of evolving systems [1]. This technique is particularly effective for studying water adsorption kinetics across different pore sizes and for monitoring gel-pore drying shrinkage [2]. Increasingly, the application of LF-NMR has been extended to alkali-activated materials (AAMs). Many researchers have adopted analytical approaches originally developed for cementitious systems, using variations in transverse relaxation times (*T*_2_) to infer pore structure evolution [3,4]. In systems such as alkali-activated metakaolin (AA-MK), this methodology may be valid due to the relatively low concentration of paramagnetic species [5]. However, in materials that contain more paramagnetic (or ferrimagnetic) species, *T*_2_ signals are significantly shortened due to paramagnetic relaxation enhancement (PRE), making such interpretations unreliable. Interestingly, despite the pronounced PRE and the paramagnetic induced signal quenching effect, *T*_2_ signals exceeding 10 ms are still observable in some ferric-rich AAM systems [6]. In highly paramagnetic matrices, the origin of these signals remains controversial, as conventional pore structure models may not be suitable to verify it. Moreover, preparing AAMs typically requires additional mixing water to ensure workability, and the excess unreacted water promotes phase separation. This can result in distinct proton populations across domains with varying solid content, each exhibiting clearly different *T*_2_ relaxation times. Therefore, broad peaks spanning 1–30 ms are commonly observed in such cases [7]. As alkali activation progresses, these peaks may shift toward longer relaxation times as free water accumulates at the top of the slurry. Such shifts have often been interpreted—using cementitious models—as evidence of capillary pore enlargement [8]. Misinterpretation of these signals can introduce significant errors in pore structure analysis and performance prediction of corresponding materials. Accurately identifying the origin of LF-NMR signals is thus essential for reliable interpretation in these systems.

In this study, we selected three materials with varying paramagnetic or ferrimagnetic content—blast furnace slag, fly ash and steel slag—and analyzed their layered *T*_2_ signals across different vertical depths after alkali activation. The selected materials span an Fe content gradient from the highest steel slag to the lowest blast furnace slag, allowing systematic evaluation of signal distortion thresholds associated with paramagnetic relaxation. In parallel, the liquid-to-solid ratios for preparing the slurry were adjusted to examine LF-NMR signal variations induced by solid-content–related heterogeneity. These results were further supported through complementary low field magnetic resonance imaging (MRI) analysis, which allows direct visualization of water distribution. The main aim of this work is to clarify the origin of LF-NMR signals in such complex, paramagnetic-rich systems and to establish practical criteria for judging when conventional interpretation models fail. Our work also demonstrates that signal shifts commonly attributed to pore-structure evolution may instead reflect physical phase separation and proposed a simple, preliminary screening method to assess slurry suitability for LF-NMR testing.

## 2. Materials and Methods

Blast furnace slag (BFS) and steel slag (SS) were sourced from a local steel plant near Beijing, while fly ash (FA) was obtained from a local power plant in Beijing. Their compositions obtained from Malvern Panalytical-Zetium XRF (Malvern Panalytical, Malvern, UK) were shown in Table 1.

Alkaline activator solutions were prepared using deionized water, consisting of 28 wt.% NaOH. The solid waste materials were activated by mixing with the NaOH solution at a liquid-to-solid ratio (L:S) of 0.45:1 (or 0.5:1). After 3 min of stirring, the resulting AAM slurry was transfer into plastic straws to a height of 5 cm using a plastic pipette. The bottom end of each straw was then sealed with Parafilm^®^. The straws were vertically positioned on a test tube rack and cured at 25 °C for 3 h until the samples set and lost their plasticity. Finally, the slurry-filled-straws were cut into five 1 cm sections using a handheld electric saw and the top, middle and bottom sections were characterized using low field MRI and NMR relaxometry. For Fe content of the top and bottom layers. Hardened paste was ground into powders, and 0.2 g of powder was added to 20 mL of 5% HNO_3_. The suspension was stirred for 30 min, centrifuged, and the supernatant was filtered through a 0.45 μm membrane. The filtrate was diluted (1:100) and analyzed for Fe content using an Agilent 5110 Synchronous Vertical Dual View (SVDV) ICP-OES (Agilent, Santa Clara, CA, USA).

**Figure 1:**
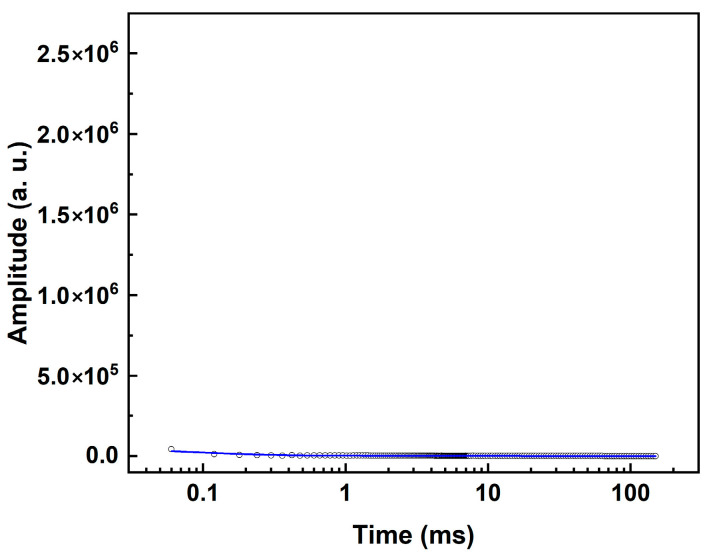
The collected relaxation signal of the empty straw.

LF-NMR relaxometry and MRI measurements were carried out at 30 °C using a 0.5 T NMR spectrometer equipped with an integrated thermostat system (Suzhou Niumag Analytical Instrument Corporation, Suzhou, China). The prepared AAM samples, still encased in plastic straws, were transferred into 10 mm glass tubes and sealed with Parafilm^®^ prior to measurement. Proton (^1^H) NMR relaxometry was performed using a 10 mm probe head with a 90° pulse length of 3 μs. Initial *T*_2_ relaxation times were acquired using the Carr-Purcell-Meiboom-Gill (CPMG) pulse sequence with a repetition delay of 1 s, 64 scans, and 2500 echoes at an echo spacing of 0.06 ms. The *T*_2_ relaxation profile was obtained by applying an inverse Laplace transform (ILT) using the Butler-Reeds-Dawson (BRD) algorithm with an alpha value of 0.1. PD-MRI measurement was performed with spin echo sequence with echo time of 4.1 ms, repetition time of 100 ms and 8 scans. To eliminate potential background signals from the plastic straws, a blank test was first conducted. As shown in Figure 1, the signal of plastic straw (wall thickness ca. 150 μm) is negligible and not enough to interfere the detection of water protons in the AAM samples. It is important to note that the XRF-derived contents of Fe and Mn oxides provide only a bulk chemical estimate of potential magnetic species. They do not directly quantify the paramagnetic moment, the strength of local magnetic field inhomogeneities, or the resultant nuclear spin relaxation rates. Future work incorporating direct magnetic measurements would be required for a quantitative correlation between composition and relaxation effects. Our study is inherently qualitative and comparative in nature. The results focus on demonstrating clear trends and phenomenological differences rather than precise quantitative metrics. Therefore, formal statistical analysis was not applied.

## 3. Results and Discussion

**Figure 2:**
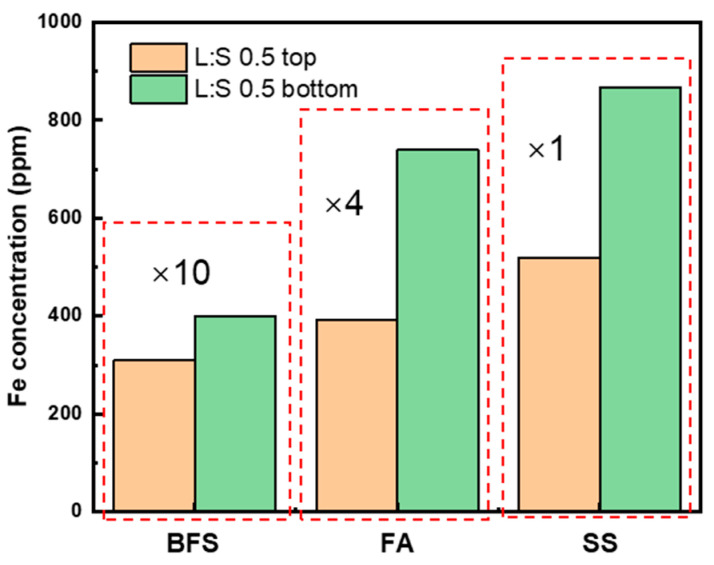
Normalized Fe concentrations of leachates from the top and bottom layers, with BFS and FA leachate concentrations scaled by factors of 10 and 4, respectively.

When characterizing cementitious or AAMs using LF-NMR, the slurry or paste is often treated as an integrated system. However, unlike homogeneous systems (e.g., solution or colloid), such systems are inherently heterogeneous. Components are unevenly distributed: denser particles tend to settle toward the bottom, while lighter components migrate toward the top. This stratification is frequently observed in relatively dilute systems, where the limited quantity of slag particles fails to effectively bind or immobilize excess water molecules. At the early stages of alkali-activation, AAM slurries primarily contain calcium- or silica-containing components (CH, SiO_2_, C_3_S, etc.), magnesium–aluminum oxides, and iron oxides. Iron oxides have densities above 5 g/cm^3^, whereas most other major calcium- or silica-containing components have densities below 3 g/cm^3^. As a result, for ferric-rich slurries with retarded setting and high workability, denser iron oxides will tend to settle during the resting period, resulting in a gradual increase in paramagnetic or ferrimagnetic substance content from the top to the bottom of the slurry (Figure 2; the leachates of AA-BFS and AA-FA are magnified.).

Such density-driven redistribution could significantly influence the LF-NMR analysis, as paramagnetic components affect the *T*_2_ relaxation behavior significantly by quenching the total signal and shorten the *T*_2_ relaxation time. Given the high concentration and potential aggregation of ferric/ferrimagnetic phases in our alkali-activated materials, the bulk magnetic susceptibility effect is the predominant cause of the rapid *T*_2_ relaxation observed.

**Figure 3:**
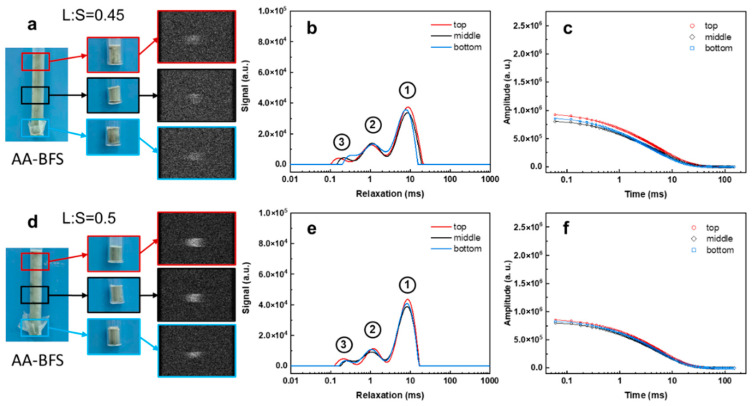
PD-MRI images (**a**,**d**), *T*_2_ relaxation profiles (**b**,**e**), and LF-NMR signal decay curves (**c**,**f**) of alkali-activated BFS at different liquid-to-solid (L:S) ratios.

To distinguish the difference in the *T*_2_ relaxation profile along vertical direction in the sample slurry, we manually sampled various vertical layers of the AAM slurry of three ferric-containing solid waste: BFS, FA and SS and analyzed them using PD-MRI and LF-NMR relaxometry. We shall start with the alkali-activated BFS (AA-BFS) slurry that has the lowest ferric content (Table 1). As shown in Figure 3, the presence of paramagnetic substances introduces distortions and artifacts in the MRI images, yielding only blurred patterns compared with the cement sample (Figure S1). Nevertheless, the signal remains detectable and sufficient to generate a PD-MRI image (echo time = 4.1 ms), indicating that an Fe_2_O_3_ + MnO content of 1.9% is insufficient to completely quench the signal. Because BFS contains a high CaO content (42.5%, Table 1) that consumes mixing water, the slurry is relatively viscous and shows minimal settling when left to stand. LF-MNR relaxation data also support this. The *T*_2_ relaxation time distribution for the top, middle, and bottom layers of the hardened BFS slurry are nearly identical, with the strongest signal component at 8–9 ms, regardless of the L:S ratio (0.45 or 0.5). The total intensity is relatively low as most of the water was consumed by the CaO (Figure 3c,f). However, as observed from the PD-MRI image the presence of paramagnetic substances hinders direct application of analysis model developed for traditional cementitious materials to AA-BFS.

**Figure 4:**
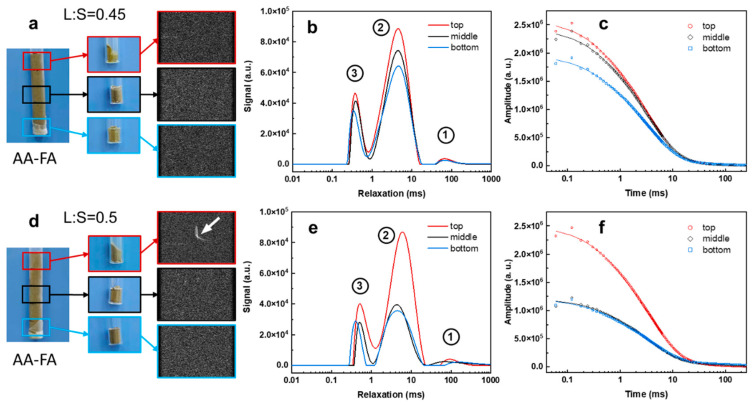
PD-MRI images (**a**,**d**), *T*_2_ relaxation profiles (**b**,**e**), and LF-NMR signal decay curves (**c**,**f**) of alkali-activated FA at different liquid-to-solid (L:S) ratios.

**Figure 5:**
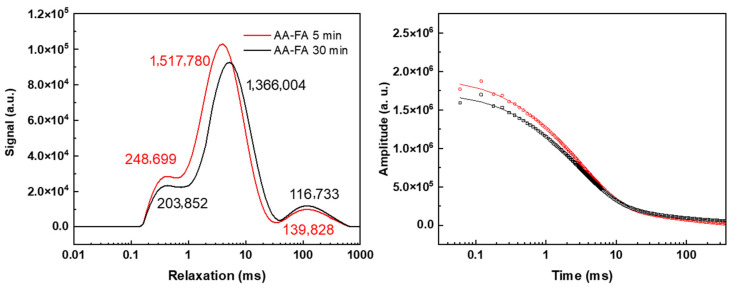
LF-NMR spectra of fresh prepared AA-FA slurry. Numbers in the figure represent the integrated of the corresponding peak.

FA contains more transition metal oxide (Fe_2_O_3_ + MnO: 3.3%) but less CaO than BFS (Table 1). Therefore, during the alkali-activation process, due to the lower content of water-absorbing CaO in alkali-activated (AA-FA) slurry, its mobile water content is significantly higher than that of AA-BFS slurry under the same L:S ratio. The resulting higher fluidity promotes the settling and separation of iron-rich components (Figure 2). As shown in Figure 4d–f, at a L:S ratio of 0.5, signal differences among the top, middle, and bottom layers become particularly pronounced. Quantitative analysis shows that the intensity of peak 2 in the top layer is approximately twice that of the bottom layer (Table S1), with a peak relaxation time of ~6 ms to about 1.5 ms longer than that of the lower layer. This is primarily due to the gravity, the solid content in FA decreases vertically from bottom to top, even after the bottom slurry had lost its plasticity, the top slurry remained flowable, indicating abundant free water. Therefore, the probability of finding a proton far away from paramagnetic-containing-grains increases from bottom to top and the signal intensities and *T*_2_ relaxation time also increases accordingly. This resulted in a shift in peak 2 toward longer relaxation in the overall spectrum (Figure 4e) after the system is settled. The relatively shorter *T*_2_ relaxation time (compared with cement paste, >80 ms) observed for protons in the top-layer free water are attributed to paramagnetic quenching. Even in freshly prepared AA-FA slurry (L:S = 0.5), the *T*_2_ relaxation times of most protons are distributed between 1 and 30 ms (Figure 5). Additionally, the total intensity of AA-FA sample is significantly higher (Figure 4c,f) than the AA-BFS samples as the net consumption of water molecules in the alkaline excitation process is very low. Consequently, originating from the low–solid-content FA suspension at the top of the AA-FA slurry, the MRI image displays a bright curve. (Figure 4d, labeled with an arrow). Therefore, in such system, an observed signal across 1 ms to 20 ms is more likely associated both with the large capillary pore water and free water in the slurry.

**Figure 6:**
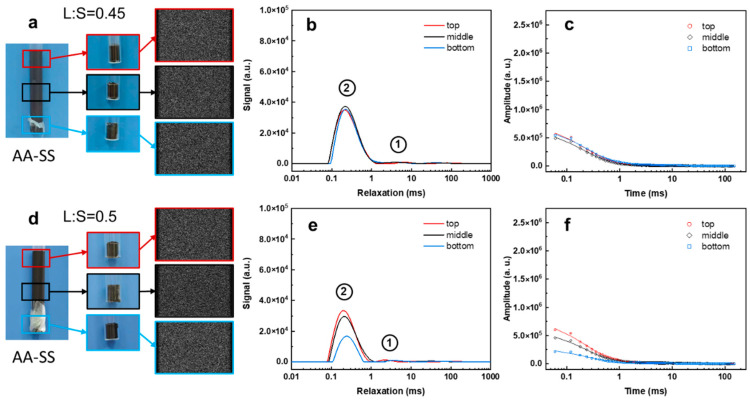
PD-MRI images (**a**,**d**), *T*_2_ relaxation profiles (**b**,**e**), and LF-NMR signal decay curves (**c**,**f**) of alkali-activated SS at different liquid-to-solid (L:S) ratios.

Among the three selected solid wastes, SS possesses the highest paramagnetic or ferrimagnetic content (Fe_3_O_4_ + MnO: 29.4%, Table 1), which markedly shortens the relaxation time of protons in free water, causing the main *T*_2_ component to appear below 1 ms. The major contributors to its strong magnetic character are expected to be ferrimagnetic Fe_3_O_4_. This is because ferrimagnetic Fe_3_O_4_ particles can have a significantly stronger effect on *T*_2_ relaxation compared to the mechanism typical of paramagnetic ions. With the echo time set to 4.1 ms, PD-MRI is unable to capture any images (Figure 6a,d). In alkali-activated slag (AA-SS) slurry with a liquid-to-solid (L:S) ratio of 0.45, the signal intensity and relaxation times remain consistent across all layers (Figure 6b,c). In contrast, at an L:S of 0.5, pronounced settling appears to occur (Figure 6e,f). However, the high paramagnetic content in AA-SS limits the reliability of LF-NMR interpretation; consequently, the obtained spectra cannot be analyzed quantitatively.

In a typical LF-NMR experiment of the whole slurry, the relaxometry data normally represent an average of the top, middle, and bottom layers. When the solid content is relatively high and the viscosity is appropriate, the results are reliable as the relaxometry data of different layers are almost consistent, yielding reliable and interpretable data. This is exemplified in studies of well-dispersed systems, where stable *T*_2_ distributions over time reflect uniform microstructural evolution [7]. Conversely, if the slurry is overly dilute and undergoes settling, the data cannot accurately reflect the proton relaxation characteristics of the system. This deviation arises from the uneven distribution of paramagnetic substances and unreacted water caused by excessive mixing water addition. This mechanism explains the commonly observed phenomenon in fly ash-rich samples during early alkali activation: the broadening of the *T*_2_ peak (1–100 ms) without a significant shift in its central position. The broadening results from the superposition of faster-relaxing signals from the paramagnetic-rich bottom layer and slower-relaxing signals from the water-rich top layer, while the average peak position remains relatively stable [9]. However, the overall solid content alone is not a reliable indicator of system stability, particularly in the presence of plasticizers or other additives, as settling (or even bleeding) can still occur even at lower L:S ratios [10]. Combining workability with free water content provides a more appropriate criterion for defining the stability threshold. However, its determination demands substantial material and time resources.

To address this, we developed a simple preliminary method to assess whether a slurry is suitable for LF-NMR testing. This method relies on diffusion of free water in the AAM slurry on filter paper, with detailed procedures provided in the Supporting Information (Section S2). It assesses the effects of workability and water-absorbing components on sedimentation but does not account for factors such as retarders, which alter the sedimentation duration. The results provide only an initial reference and require further validation through comprehensive experimental studies. Accordingly, the method is not discussed in detail in this paper.

## 4. Conclusions

In alkali-activated systems, especially those with relatively high paramagnetic content, particle settling can severely compromise LF-NMR data reliability. Therefore, factors such as the content of paramagnetic substances, the content of water-consuming components, the presence of plasticizers, and the liquid-to-solid ratio must be carefully considered before measurement. The following conclusions can be drawn from the results:

1. In relatively dilute systems, settlement increases the free water content in the top slurry layer and reduces the concentration of paramagnetic substances, while the lower layer exhibits the opposite trend. As curing time extends, longer proton relaxation times may be observed in the LF-NMR spectrum as the signals from accumulated free water in the top layer slurry become dominant.

2. Any additive or condition that can prevent settling of the slurry will benefit the accurate interpretation of the LF-NMR data. Therefore, BFS slurries with high CaO (>40%) content showed minimal settling and consistent relaxation profiles, allowing meaningful interpretation once standard interpretation model is established. In contrast, FA slurries exhibited strong stratification at higher liquid-to-solid ratios, producing vertical signal gradients that render LF-NMR analysis unreliable. This is similar to how soil retains water whereas sand does not.

3. Vertical homogeneity of unreacted water is a key criterion to identify whether the AAM slurry is suitable for LF-NMR characterization. A simple screening method is proposed in this work to rapidly evaluate slurry homogeneity, though further validation is required.

## Figures and Tables

**Table 1:** Chemical compositions (in wt%) of the fly ash, blast furnace slag and steel slag.

Compositions	BFS	FA	SS
**CaO**	42.5	28.3	37.1
SiO_2_	29.5	33.8	15.5
Al_2_O_3_	13.8	16.5	7
**Fe_3_O_4_**	--	--	27.7
**Fe_2_O_3_**	1.7	3.1	--
P_2_O_5_	--	0.6	1.9
MgO	7.7	8.9	7.4
SO_3_	2.2	4.2	--
Na_2_O	0.5	1.9	--
**MnO**	0.2	0.2	1.7
Others	1.9	2.5	1.7

## Data Availability

The original contributions presented in this study are included in the article/Supplementary Materials. Further inquiries can be directed to the corresponding authors.

## Supplementary Materials

The following supporting information can be downloaded at https://www.mdpi.com/article/10.3390/ma19020272/s1, Figure S1. PD-MRI image of (a) cement paste with L:S of 0.40 and (b) AA-BFS with L:S of 0.50. Figure S2. Schematic illustration for the assessment for vertical water distribution in the slurry. Figure S3. LF-NMR spectra and signal decay curves of alkali-activated FA: (a,b) L:S = 0.45 sample classified as negative by the diffusion method; (c,d) L:S = 0.5 sample classified as positive by the diffusion method. Table S1. Integration areas and weight-normalized LF-NMR peak Figure 1, Figure 2 and Figure 3.

## Abbreviations

The following abbreviations are used in this manuscript:

BFS	blast furnace slag
FA	fly ash
SS	steel slag
AAMs	alkali-activated materials
MK	metakaolin
LF-NMR	low-field nuclear magnetic resonance
CPMG	Carr-Purcell-Meiboom-Gill
BRD	Butler-Reeds-Dawson
ILT	inverse Laplace transform
PD-MRI	proton density magnetic resonance imaging
CH	calcium hydroxide
C_3_S	tricalcium silicate
