# Supplementary material for: The Influence of Solid Content Distribution on the Low-Field Nuclear Magnetic Resonance Characterization of Ferric-Containing Alkali-Activated Materials
Source: Materials (Basel). 2026 Jan 9;19(2):272. doi: 10.3390/ma19020272 (PMC12842970; doi:10.3390/ma19020272)
Supplement: Supplementary file 1 [file materials-19-00272-s001.zip › materials-4082702-supplementary.pdf]

The influence of solid content distribution on the low-field nuclear magnetic resonance characterization of ferric-containing alkali-activated materials

Zian Tang [1,2,*], Yuanrui Song [1,2], Wenyu Li [3,*], Lingling Zhang [1,2]

1. School of Energy and Environmental Engineering, University of Science and Technology Beijing, Beijing 100083, China.
2. State Key Laboratory of Iron and Steel Industry Environmental Protection, University of Science and Technology Beijing, Beijing, China.
3. Department of Chemistry, Tsinghua University, Beijing 100084, China

* Corresponding authors.

E-mail addresses: ziantang@ustb.edu.cn (Z. Tang), wenyuli@mail.tsinghua.edu.cn (W. Li).

S1. Comparison of PD-MRI Images of Cement Paste and AA-BFS Slurry

The PD-MRI image of the cement paste (L:S=0.4) was obtained using the same method as described in the experimental section.

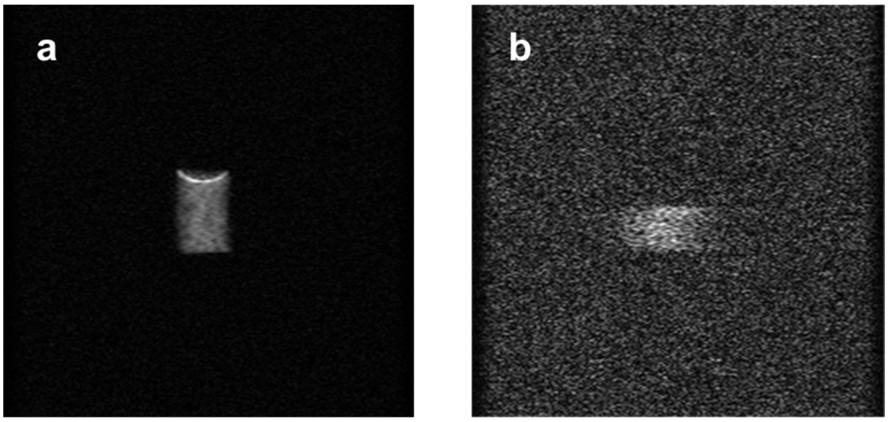

Figure S1. PD-MRI image of a) cement paste with L:S of 0.40 and b) AA-BFS with L:S of 0.50

S2. Water content evaluation

Slurry water distribution was assessed using the filter paper diffusion method (experimental setup in Figure S2). Filter papers were sprayed with phenolphthalein solution and dried at 60 °C for 30 min (Figure S2a). Stirred slurry was transferred into a plastic cup to a height of 4 cm, sealed, and allowed to stand for 5 min. Samples of 0.1 mL were collected from the top and bottom layers using 1 mL syringes individually, avoiding bubble formation (Figure S2b). Afterwards, each syringe was held vertically, with the outlet positioned at the center of the filter paper, allowing the slurry to contact

the filter paper by gravity (Figure S2c). After 10 s contact, the syringe was removed from the filter paper and the diameter of the liquid halo was recorded (Figure S2d). The test was repeated three times for each layer, yielding top-layer diffusion distance and bottom-layer diffusion distance. Data were analyzed using an independent-samples t-test (90% confidence level). A statistically significant difference between the two "distance sets" indicated uneven distribution of the water content, yielding a "positive" result and deeming the slurry unsuitable for LF-NMR testing. Conversely, the absence of a significant difference yielded a "negative" result, confirming the suitability.

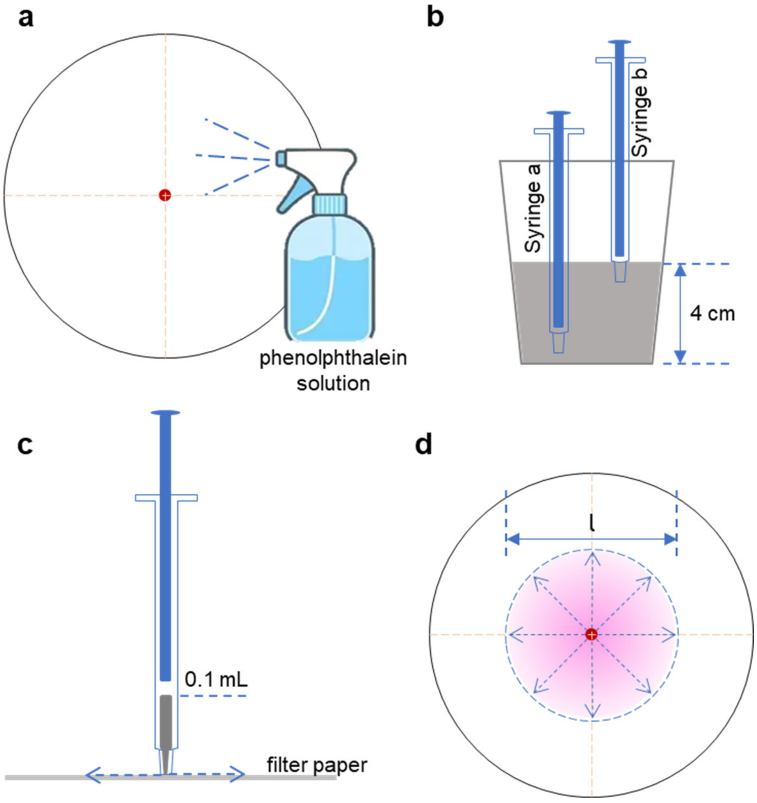

Figure S2. Schematic illustration for the assessment for vertical water distribution in the slurry.

This diffusion-based method was validated with AA-FA (from a different FA source than that used in the main text), and the corresponding LF-NMR results are shown in Figure S3. As demonstrated, the sample classified as negative exhibits similar LF-NMR signals in its top and bottom layers (Figure S3a), In contrast, the positive sample exhibits a top layer enriched in protons with T_2 values exceeding 20 ms, while

containing fewer protons with T_2 values between 1 and 20 ms as the bottom layer (Figure S3c).

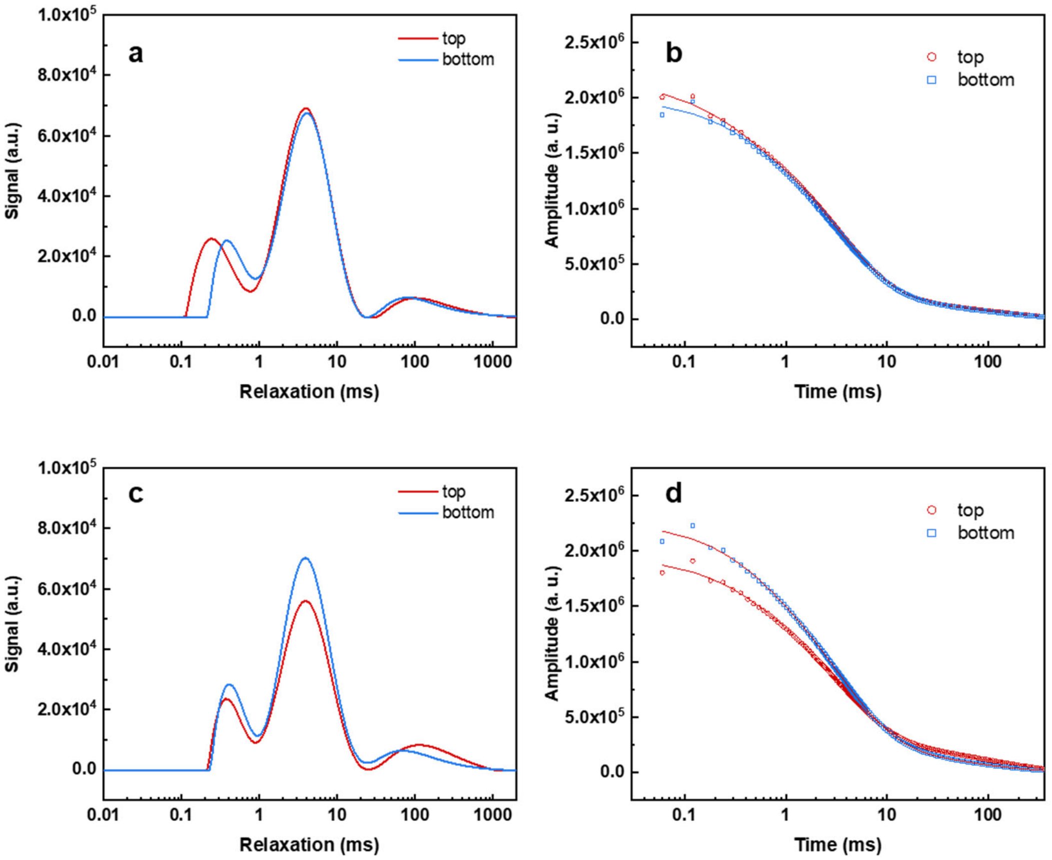

Figure S3. LF-NMR spectra and signal decay curves of alkali-activated FA: (a, b) L:S = 0.45 sample classified as negative by the diffusion method; (c, d) L:S = 0.5 sample classified as positive by the diffusion method.

S3. Integration area of LF-NMR peaks

The integration areas of the LF-NMR peaks and their normalized values within each solid waste type are summarized in Table S1. Intensities were normalized to sample weight.

Table S1. Integration areas and weight-normalized LF-NMR peak Figure 1-3.

Sample name	location	Peak 1	Peak 2	Peak3
AA-BFS L:S 0.45	top	674701	238078	47520
	middle	570761	240306	44602
	bottom	583722	257240	47422
AA-BFS L:S 0.5	top	663179	163500	57927
	middle	625729	160446	34940
	bottom	641583	176372	35055
AA-FA L:S 0.45	top	60353	2071793	448694
	middle	46879	1713842	389949
	bottom	56588	1570966	333235
AA-FA L:S 0.5	top	58596	1956088	481545
	middle	77836	892483	232192
	bottom	69526	886675	248186
AA-SS L:S 0.45	top	20871	733764	N.A.
	middle	27503	777235	N.A.
	bottom	27401	688581	N.A.
AA-SS L:S 0.5	top	26382	645977	N.A.
	middle	26205	586354	N.A.
	bottom	22967	263748	N.A.
